# Composite Meshes: A Comprehensive Review of Materials, Biomechanics, Clinical Outcomes, and Emerging Innovations

**DOI:** 10.7759/cureus.107431

**Published:** 2026-04-20

**Authors:** Monika Joshi, Nitish Jhawar

**Affiliations:** 1 General Surgery, Apollo Hospitals, Mumbai, IND; 2 General and Colorectal Surgery, Apollo Hospitals, Mumbai, IND

**Keywords:** adhesion prevention, barrier layer, biomechanics, composite mesh, expanded polytetrafluoroethylene, hernia repair, inguinal hernia, laparoscopic ventral hernia repair, mesh classification, polypropylene

## Abstract

Hernia repair is among the most frequently performed surgical procedures worldwide. Composite meshes, that is, prostheses incorporating at least two distinct functional layers, were developed to reconcile the competing demands of structural durability and peritoneal biocompatibility. This narrative review synthesizes evidence from peer-reviewed research articles, physicomechanical evaluation studies, and clinical outcome data identified through PubMed, MEDLINE, and Scopus searches spanning 2000-2025. Composite meshes are broadly classified by barrier type as either absorbable or permanent (nonabsorbable). Key physicochemical parameters, namely, pore size, filament diameter, mesh weight, suture retention strength, tear resistance, and anisotropy, significantly influence host tissue integration and clinical performance. Absorbable barrier meshes, including C-QUR (Atrium Medical Corporation, Hudson, New Hampshire, United States), PROCEED (Ethicon, Inc. (a Johnson & Johnson company), Somerville, New Jersey, United States), Parietex Composite (Sofradim Production SAS (a Medtronic company), Trévoux, France), and Bard Sepramesh IP Composite (Davol Inc. (a subsidiary of C.R. Bard, Inc., now Becton, Dickinson and Company), Warwick, Rhode Island, United States), facilitate eventual tissue incorporation after barrier degradation within 30-120 days. Permanent barrier meshes, including DUALMESH (W.L. Gore & Associates, Inc., Flagstaff, Arizona, United States) and Bard Composix E/X and L/P (Davol Inc. (a subsidiary of C.R. Bard, Inc., now Becton, Dickinson and Company), Warwick, Rhode Island, United States), provide durable adhesion resistance through an intact expanded polytetrafluoroethylene surface. Clinically, composite meshes demonstrate recurrence rates comparable to conventional polypropylene with measurably reduced adhesion formation in the intraperitoneal position. Complications, including chronic pain, seroma formation, mesh shrinkage, infection, and rare visceral erosion, remain pertinent concerns. Future innovations, including drug-eluting coatings, auxetic mesh geometries, patient-specific three-dimensional printing, and biodegradable biosynthetic scaffolds, hold considerable promise in further optimizing surgical outcomes.

## Introduction and background

Introduction

A hernia is defined as the protrusion of an organ or tissue through a defect in the surrounding body wall. Abdominal wall hernias, encompassing the most commonly encountered subtypes, including inguinal, femoral, umbilical, and incisional hernias, represent a major global health burden. Inguinal hernias account for approximately 70-75% of all hernias, with femoral (6-17%) and umbilical (3-8.5%) variants comprising much of the remainder [1]. Hernia repair has emerged as one of the most common elective general surgical procedures globally, with prosthetic biomaterials continuing to evolve in parallel with advances in operative technique [2].

Prior to the widespread adoption of mesh-based repair, hernia surgery relied on suture-based techniques that carried recurrence rates approaching 10-15% for primary inguinal hernias and exceeding 30% for incisional hernias [3,4]. The introduction of tension-free mesh repair by Lichtenstein et al. represented a significant advance in hernia surgery, with subsequent studies demonstrating a marked reduction in recurrence rates and improved postoperative recovery compared with tissue-based repairs [5]. Nearly 150 distinct biomaterial designs are now available for hernia repair applications [6], reflecting the breadth of ongoing innovation in this field.

Despite the success of simple synthetic meshes, their use in the intraperitoneal placement (positioning of the mesh within the abdominal cavity, in direct contact with the visceral organs) carries well-documented risks. Direct contact between standard polypropylene mesh and visceral structures is associated with dense adhesion formation and, at reoperation, a small bowel resection rate of 20% for non-barrier meshes [7]. This limitation catalyzed the development of composite meshes: multi-layer prostheses designed to provide durable abdominal wall reinforcement on one surface while protecting underlying viscera on the other. This review provides a comprehensive evaluation of composite meshes for abdominal and inguinal hernia repair, encompassing their classification, material composition, physicochemical properties (the combined physical and chemical characteristics of the material, including porosity, tensile strength, and surface reactivity), tissue-mesh interface biomechanics, clinical outcomes, complications, and future directions.

Historical background

The history of prosthetic hernia repair reflects a progressive refinement in material science and surgical philosophy. Theodor Billroth's visionary proposal in 1857, that artificially produced tissue of fascia-like density could cure hernias, foreshadowed modern mesh repair [8]. It was not until 1940 that Burke introduced tantalum metal sheets closely resembling contemporary prostheses, though severe complications prompted the search for alternatives. Nylon was among the first plastics woven into mesh, but its susceptibility to hydrolytic degradation led to failure. The search for a non-metallic, nonabsorbable synthetic resistant to infection guided the development of polypropylene, polytetrafluoroethylene (PTFE), polyester, and related materials [8].

Since the introduction of polypropylene mesh by Usher et al. in the early 1960s, these four material groups have formed the backbone of prosthetic hernia repair. The field has since been driven by the recognition that no single mesh fulfils all ideal criteria [9]. Modifications to conventional mesh design, including adaptation of pore architecture, filament diameter, and overall density to the mechanical demands of the abdominal wall, were subsequently proposed to optimize the tissue-prosthesis interface and reduce inflammatory burden [10]. These modifications laid the conceptual groundwork for the lightweight and composite mesh generation that followed.

The intraperitoneal use of standard polypropylene mesh became problematic as laparoscopic techniques gained favor; direct visceral contact was associated with dense adhesion formation, which drove the engineering of composite constructs pairing a structural reinforcing layer with an anti-adhesive barrier [7,8].

Methods

Search Strategy

**Figure 1:**
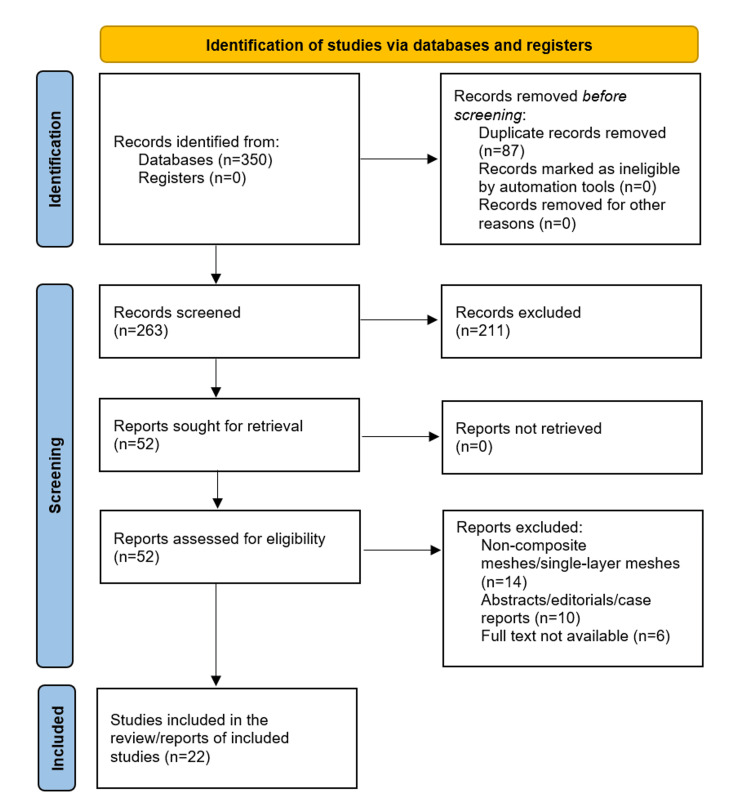
PRISMA flowchart PRISMA: Preferred Reporting Items for Systematic Reviews and Meta-Analyses

This narrative review was conducted in accordance with the Preferred Reporting Items for Systematic Reviews and Meta-Analyses (PRISMA) 2020 reporting guidelines (Figure 1). A systematic search of the PubMed/MEDLINE, Scopus, and Web of Science Core Collection electronic databases was performed for the period January 2000 to December 2025. The following Medical Subject Headings (MeSH) terms and free-text keywords were used in combination using Boolean operators: "composite mesh", "hernia mesh", "hernia repair", "polypropylene mesh", "expanded polytetrafluoroethylene", "barrier mesh", "intraperitoneal mesh", "mesh biomechanics", "physicochemical properties mesh", "mesh adhesion", "laparoscopic ventral hernia repair", "inguinal hernia repair", and "mesh complications". Reference lists of included articles were hand-searched to identify additional eligible sources. 

Inclusion and Exclusion Criteria

Studies were considered eligible for inclusion if they (1) reported on composite prostheses used in abdominal or inguinal hernia repair, (2) provided data on physicochemical properties, biomechanical performance, clinical outcomes, or complication profiles of composite meshes, (3) were published in English in peer-reviewed journals, and (4) were published within the period 2000-2025. Historical studies published before 2000 were selectively included where they represented foundational or seminal contributions to the field (e.g., Lichtenstein et al. [5] and Amid [11]). Studies were excluded if they (1) reported exclusively on non-composite (single-layer) synthetic meshes without a barrier component, (2) were conference abstracts, editorials, or case reports without systematic data, or (3) were not available in full text.

Study Selection

Initial screening of titles and abstracts was performed independently by both authors. Full-text review was subsequently conducted for all potentially eligible records. Disagreements were resolved by consensus. A total of 22 references are cited in this review, encompassing original research articles, systematic reviews, randomized controlled trials, and validated biomechanical evaluation studies. 

## Review

Classification of composite meshes

Composite meshes are broadly defined as prostheses composed of two or more distinct material layers, each serving a separate biological or mechanical function. Deeken et al. proposed a hierarchical classification system beginning with the structural mesh component and further stratifying based on additional modifying layers [12]. A parallel classification was described by Amid based on pore architecture, ranging from macroporous (type I) to microporous (type IV) [11]. In composite barrier meshes, two discrete layers are pressed or sewn together: the structural mesh, oriented toward the abdominal wall, and an anti-adhesion barrier, oriented toward the viscera.

Titanium-coated polypropylene meshes have attracted interest as a strategy to attenuate chronic postoperative inguinal pain. Narrative reviews of titanized mesh in inguinal repair have demonstrated that while recurrence rates are comparably low, the evidence base for superiority over conventional polypropylene remains insufficient [13]. Mechanisms underlying chronic pain, namely, foreign body reaction, perineural inflammation, and mesh-related fibrosis, appear to be more attenuated by reduced mesh density and favorable pore architecture rather than surface coating modification alone [14,15].

Absorbable barrier meshes incorporate a degradable layer that temporarily prevents visceral adhesions during the critical early postoperative period. Most absorbable barriers degrade within 30-120 days. Reperitonealization typically occurs within five to seven days, so clinically significant adhesions are generally not expected to form once the barrier has been absorbed [12]. Permanent (nonabsorbable) barrier meshes employ a durable anti-adhesive surface, most commonly expanded PTFE (ePTFE), that persists indefinitely, providing sustained visceral protection at the cost of limiting tissue ingrowth on the barrier side.

An expanded and updated classification framework proposed by Deeken and Lake in 2017 stratifies hernia repair biomaterials into three principal structural categories: permanent synthetic (encompassing polypropylene, ePTFE, and polyester-based constructs), resorbable synthetic (including poly-4-hydroxybutyrate and polyglycolide-based materials), and biological tissue-derived (encompassing human, porcine, and bovine acellular dermal matrices and submucosa-derived scaffolds) [6]. Within each top-level category, meshes are further subdivided into bare meshes, those incorporating anti-adhesion barriers or surface coatings, and reinforced constructs in which supplementary fibers augment mechanical performance [6]. As of 2017, nearly 150 distinct biomaterial designs had been catalogued within this system, reflecting the substantial commercial breadth and ongoing innovation in hernia prosthetics, and the framework supersedes earlier pore-based classifications by providing a hierarchical, composition-centered reference applicable across open and laparoscopic approaches [6]. The classification, composition, and key physicochemical properties of representative composite meshes evaluated for laparoscopic ventral hernia repair are presented in Table 1.

**Table 1:** Classification, composition, and key properties of composite hernia meshes evaluated for laparoscopic ventral hernia repair PP: polypropylene; PDS: polydioxanone; ORC: oxidized regenerated cellulose; PGA: polyglycolic acid; HA: hyaluronic acid; CMC: carboxymethylcellulose; PEG: polyethylene glycol; PET: polyethylene terephthalate; ePTFE: expanded polytetrafluoroethylene Table adapted from Deeken et al. [12]

Mesh product	Structural layer	Barrier/coating	Key physicochemical properties
C-QUR (Atrium Medical Corporation, Hudson, New Hampshire, United States)	PP	Omega-3 fatty acid gel (absorbable, >120 days)	Medium interstices; medium weight post-absorption; physiologic strain below range
PROCEED (Ethicon, Inc. (a Johnson & Johnson company), Somerville, New Jersey, United States)	PP encapsulated in PDS	ORC (absorbable)	Very large interstices; ultra-small filament diameter; ball burst >50 N/cm
Bard Sepramesh IP Composite (Davol Inc. (a subsidiary of C.R. Bard, Inc., now Becton, Dickinson and Company), Warwick, Rhode Island, United States)	PP co-knitted with PGA	HA/CMC/PEG hydrogel (absorbable)	Highest suture retention of absorbable group (99.2 N parallel); medium interstices
Parietex Composite (Sofradim Production SAS (a Medtronic company), Trévoux, France)	PET	Type I collagen layer (absorbable)	Very large interstices; lowest ball burst strength (38.9 N/cm)
Bard Composix E/X (Davol Inc. (a subsidiary of C.R. Bard, Inc., now Becton, Dickinson and Company), Warwick, Rhode Island, United States)	PP (heavy weight)	ePTFE (permanent)	Medium interstices; physiologic strain range; greatest lateral anisotropy
Bard Composix L/P (Davol Inc. (a subsidiary of C.R. Bard, Inc., now Becton, Dickinson and Company), Warwick, Rhode Island, United States)	PP (light weight)	ePTFE (permanent)	Very large interstices; physiologic strain range; light PP component
DUALMESH Biomaterial (W.L. Gore & Associates, Inc., Flagstaff, Arizona, United States)	ePTFE	Smooth ePTFE surface (permanent)	Microporous; greatest density (420 g/m²); physiologic strain range; infection risk

Material composition of composite meshes

Structural Layer Materials

Polypropylene remains the dominant structural material in composite mesh design. A hydrophobic carbon polymer with alternating methyl moieties, polypropylene is flexible, highly resistant to infection, and readily integrated by surrounding tissues through its large-pore monofilament architecture [8]. Its monofilament structure facilitates fibrovascular ingrowth, resists infection more effectively than multifilament designs, and supports tissue incorporation, the principal goal for the structural layer of any composite prosthesis. Polypropylene remains the most popular material in mesh hernia repair globally [8].

ePTFE is characterized by high electronegativity, chemical inertness, and pronounced hydrophobicity. These properties limit tissue adhesion, making ePTFE well-suited as a visceral-facing barrier. Experimental studies have demonstrated the formation of a well-organized neoperitoneum, with parallel collagen fibers covered by mesothelial cells, on the peritoneal-facing surface of ePTFE implants, effectively preventing visceral adhesion [2]. However, the microporous architecture of PTFE allows bacterial passage while preventing macrophage penetration, increasing infection risk and virtually always necessitating mesh explantation when infection occurs [8].

Polyethylene terephthalate (PET) or polyester is a hydrophilic polymer fashioned into strong fibers suitable for weaving into multifilament mesh. Its hydrophilic nature promotes cell adhesion and tissue ingrowth, and it tends to result in minimal adhesion formation to adjacent organs. Parietex Composite (Sofradim Production SAS (a Medtronic company), Trévoux, France) employs a PET structural layer combined with an absorbable collagen barrier. However, polyester is susceptible to hydrolytic degradation over time, raising concerns about long-term mechanical stability compared with polypropylene or ePTFE [8].

Barrier and Coating Materials

A diverse range of materials has been employed as anti-adhesion barriers. Omega-3 fatty acid gel coatings, derived from fish oil, coat the C-QUR mesh (Atrium Medical Corporation, Hudson, New Hampshire, United States) and are absorbed over more than 120 days. Hydrogel barriers composed of sodium hyaluronate, carboxymethylcellulose, and polyethylene glycol (as in Sepramesh IP) exploit the viscoelastic and lubricating properties of these biopolymers. Oxidized regenerated cellulose forms a conformable film in PROCEED mesh (Ethicon, Inc. (a Johnson & Johnson company), Somerville, New Jersey, United States). Type I collagen coatings in Parietex Composite create a transient peritoneal interface layer with native extracellular matrix biocompatibility. Resorbable synthetic filaments, including polyglactin 910 and polyglecaprone, have been co-knitted with polypropylene to create partially absorbable hybrid constructs such as Vypro II and Ultrapro, respectively, reducing post-absorption mesh mass while retaining permanent polypropylene reinforcement [2].

Physicochemical and Biomechanical Properties

Understanding the physicochemical properties of composite meshes is essential for appropriate clinical selection. Deeken et al. performed comprehensive biomechanical testing of seven composite prostheses for laparoscopic ventral hernia repair using standardized American Society for Testing and Materials (ASTM) protocols, providing the most rigorous comparative dataset available [12]. The results of this testing are presented in the figures below.

Standardized biomechanical testing protocols underpin rigorous composite mesh characterization [6]. In suture retention testing, a stainless steel wire is passed through the mesh at a fixed distance from the edge to simulate a suture, and the maximum load sustained prior to failure at that fixation point defines suture retention strength. Tear resistance testing initiates a small controlled incision and records the maximum load sustained before progressive tear propagation. Ball burst testing applies compressive force via a stainless steel ball, simulating intra-abdominal pressure loading; given that the maximum physiologically relevant tensile stress at extreme intra-abdominal pressure and abdominal circumference approaches 47.8 N/cm, a minimum ball burst strength of 50 N/cm has been recommended for hernia repair materials [6]. Biaxial tensile testing simultaneously loads the mesh in two orthogonal directions, more faithfully replicating the multiaxial physiological stress environment of the abdominal wall than uniaxial testing alone; this method also enables the quantification of anisotropy through a stiffness-based anisotropy index [6]. Collectively, the recommended minimum failure properties for composite meshes are suture retention strength and tear resistance both exceeding 20 N, ball burst strength exceeding 50 N/cm, and physiologic strain within the 10-30% range observed in the native abdominal wall [6].

Pore Size and Filament Diameter

Pore size critically determines the biological behavior of an implanted mesh. Macroporous meshes with interstice dimensions exceeding 1 mm allow passage of macrophages, fibroblasts, blood vessels, and collagen fibers, promoting tissue ingrowth and mechanical anchorage to the abdominal wall [12]. Meshes with interstice dimensions below 1 mm may result in scar plate bridging between filaments and the encapsulation of the entire mesh rather than genuine tissue integration, contributing to a foreign body sensation [14]. Microporous meshes such as DUALMESH (W.L. Gore & Associates, Inc., Flagstaff, Arizona, United States) harbor bacteria while excluding macrophages, increasing infection susceptibility and predisposing to seroma formation due to impaired fluid clearance [12].

Filament diameter modulates both the surface area in contact with host tissue and the flexural rigidity of the mesh. Bending stiffness is related to the fourth power of filament diameter: halving filament diameter results in approximately a 16-fold increase in flexibility [12]. Reducing filament diameter thus substantially lowers the foreign body burden while improving mesh conformability to abdominal wall motion during respiration and activity.

Mesh Weight Classification

Composite meshes have been classified by weight density, with direct implications for tissue response and clinical performance, as summarized in Table 2.

**Table 2:** Mesh weight classification and clinical implications for hernia repair Table adapted from Klosterhalfen et al. [15] and Rodríguez et al. [2]

Weight class	Density	Typical pore size	Clinical implications
Heavyweight	>90 g/m²	<1 mm	Higher tensile strength; greater foreign body response; abdominal wall stiffening; increased chronic pain risk
Medium weight	50-90 g/m²	1-2 mm	Balanced tissue integration and structural support; intermediate inflammatory response
Lightweight	35-50 g/m²	>1 mm	Reduced chronic inflammation; improved compliance; preferred for inguinal hernia repair
Ultra-lightweight	<35 g/m²	>2 mm	Minimal foreign body reaction; risk of structural failure under high intra-abdominal pressures

Mesh weight classification has evolved over several decades. Klosterhalfen et al. proposed the lightweight and large porous mesh concept, demonstrating that reduction of mesh density below 50 g/m² substantially reduces the foreign body burden and inflammatory response while maintaining adequate structural support [15]. Stereographic studies by Welty et al. documented that increased mesh density correlates directly with reduced abdominal wall compliance and greater patient-reported pain and restricted movement [16]. For inguinal hernia repair, lightweight, large-pore meshes are now preferred, offering better flexibility and reduced chronic pain without increasing recurrence rates [15].

Suture Retention Strength and Tear Resistance

**Figure 2:**
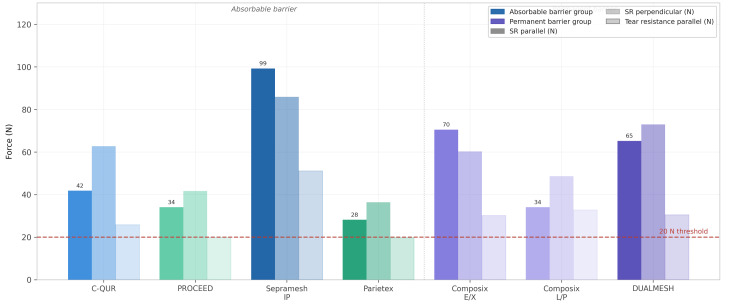
Suture retention strength and tear resistance of composite meshes Grouped bar chart comparing suture retention strength (parallel and perpendicular, N) and tear resistance (parallel, N) for seven composite mesh prostheses. Dashed red reference line indicates the minimum threshold of 20 N. All meshes exceed this threshold. Bard Sepramesh IP Composite demonstrates the highest parallel suture retention (99.2 N). All values represent mean±SEM. Figure adapted from Deeken et al. [12] and created using GraphPad Prism (GraphPad Software, Boston, Massachusetts, United States)

Suture retention strength represents the maximum load a mesh can withstand at a fixation point before the suture tears through the material. Animal model studies have demonstrated that zero-polypropylene sutures provide approximately 20-30 N of resistance per suture when attached to porcine abdominal wall tissue, establishing a minimum clinical benchmark [12]. In the comparative evaluation by Deeken et al., all seven composite meshes exceeded 20 N suture retention in both parallel and perpendicular orientations [12]. Among absorbable barrier meshes, Bard Sepramesh IP Composite (Davol Inc. (a subsidiary of C.R. Bard, Inc., now Becton, Dickinson and Company), Warwick, Rhode Island, United States) demonstrated the greatest parallel suture retention at 99.2 N, more than double the second-highest value in that group. Among permanent barrier meshes, DUALMESH led in the perpendicular direction (Figure 2).

Tear resistance reflects the capacity of a mesh to resist progressive tearing once initiated, a clinically relevant scenario when tacks or sutures concentrate stress during sudden intra-abdominal pressure increases such as during coughing or sneezing. Parietex Composite, PROCEED, and Bard Composix L/P (Davol Inc. (a subsidiary of C.R. Bard, Inc., now Becton, Dickinson and Company), Warwick, Rhode Island, United States) exhibited tear strengths below 20 N in at least one testing direction [12], suggesting potential vulnerability to propagation tears under high physiologic loading.

Tensile Strength, Ball Burst, and Anisotropy

**Figure 3:**
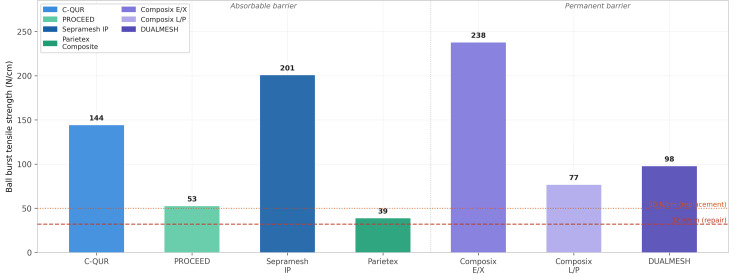
Ball burst tensile strength of composite meshes Ball burst tensile strength (N/cm) for seven composite mesh prostheses. Red dashed line (32 N/cm) denotes the minimum threshold for abdominal wall repair; orange dotted line (50 N/cm) denotes the threshold for abdominal wall replacement. All meshes except Parietex Composite exceed 50 N/cm. Bard Composix E/X demonstrates the highest ball burst strength (237.8 N/cm). BMI: body mass index Figure adapted from Deeken et al. [12] and created using GraphPad Prism (GraphPad Software, Boston, Massachusetts, United States)

Theoretical tensile stress requirements for hernia mesh repair range from 10.5 N/cm for a spherical model in a normal-weight woman with an intra-abdominal pressure of 20 kPa to 47.8 N/cm for a cylindrical model in an obese man with an intra-abdominal pressure of 30 kPa [12]. Normal healthy adults generate maximum intra-abdominal pressures up to 23 kPa during activities such as jumping [17]. All seven composite meshes demonstrated ball burst tensile strengths exceeding 32 N/cm; all except Parietex Composite exceeded 50 N/cm (Figure 3) [12].

**Figure 4:**
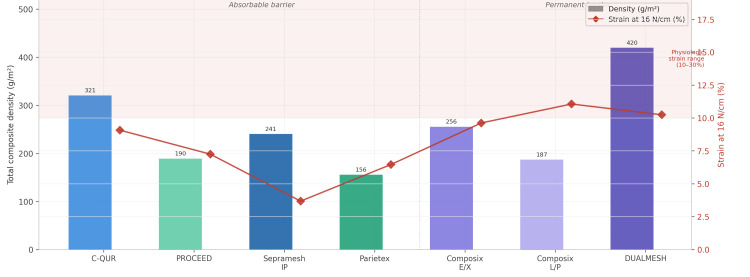
Mesh density and physiologic strain comparison Dual-axis comparison of total composite mesh density (bars, g/m², left axis) and strain at 16 N/cm (diamonds, %, right axis). The shaded zone indicates the physiologically relevant strain range of the human anterior abdominal wall (10-30%). Only Bard Composix E/X, Bard Composix L/P, and DUALMESH achieve physiologic strain values. Figure adapted from Deeken et al. [12] and Junge et al. [18] and created using GraphPad Prism (GraphPad Software, Boston, Massachusetts, United States)

A frequently overlooked property is anisotropy, the dependence of mechanical behavior on testing direction. Most composite meshes exhibit significantly different properties when tested parallel versus perpendicular to the longest mesh dimension [12]. The most compliant axis of the biomaterial should be oriented cranio-caudally (longitudinal), and the strongest axis should be oriented transversely (medial-lateral), as the human abdominal wall experiences greater transverse stresses [6]. At a physiologically relevant stress of 16 N/cm, only Bard Composix E/X (Davol Inc. (a subsidiary of C.R. Bard, Inc., now Becton, Dickinson and Company), Warwick, Rhode Island, United States), Bard Composix L/P, and DUALMESH achieved strain values within the physiologic range of 10-30% reported for the human anterior abdominal wall [18]; the remaining meshes demonstrated strain values below 10%, potentially limiting synchronous movement with the abdominal wall (Figure 4).

Abdominal Wall Biomechanics and the Mesh-Tissue Interface

The human abdominal wall is a complex, layered, anisotropic composite structure. The linea alba comprises a three-dimensional meshwork of type I collagen exhibiting sequential intermingling oblique layers, predominantly transverse fibers, and variable irregular fibers [6]. It demonstrates greater compliance in the cranio-caudal direction and greater stiffness transversally, with anisotropy ratios as high as 8 to 9 [6]. Current composite mesh designs exhibit anisotropy ratios of only 1 to 3, a fundamental mechanical mismatch with native tissue [6].

Quantitative mechanical characterization of individual abdominal wall tissue layers has provided important benchmarks for biomaterial design [6]. Uniaxial and biaxial tensile testing of the linea alba has reported transverse elastic moduli of approximately 70-80 kPa compared with longitudinal moduli of 8-32 kPa, with transverse stresses consistently 2-3 times greater than longitudinal values across multiple independent cohorts [6]. In multidirectional ex vivo testing at a reference load of 16 N, the intact abdominal wall exhibits greatest strain in the cranio-caudal direction (approximately 27%), followed by the medial-lateral (approximately 16%) and oblique directions (approximately 12%), with female specimens demonstrating increased compliance compared with males [6]. Biaxial tensile studies further demonstrate that linea alba mechanics are approximately 30% stronger than incisional scar tissue, providing mechanistic insight into heightened susceptibility to incisional hernia following midline laparotomy [6]. External abdominal surface strains have been found to be approximately twice those measured at the internal surface, suggesting that extrapolation of internal strains from external measurements alone may be unreliable in the clinical setting [6]. Anisotropy ratios across studies of the linea alba range from approximately 2 to 14.5, reflecting heterogeneity in methodology and specimen preparation, and underscore that a single universal anisotropy target for mesh design cannot be established without further standardized investigation [6].

This mismatch is clinically significant. Most hernia recurrences occur not from structural mesh failure but from failure at the biomaterial-tissue interface, driven by insufficient mesh-defect overlap, poor tissue integration, sustained inflammatory contracture, or migration from fixation failure [6]. Deeken and Lake comprehensively reviewed this discrepancy and concluded that no single current biomaterial design fully encompasses all ideal mechanical features [6].

Finite element analysis (FEA) has emerged as a valuable computational complement to experimental testing. By simulating mesh-tissue interactions under physiological intra-abdominal pressure loading and varying defect sizes and locations, FEA enables the optimization of mesh mechanical properties before clinical deployment [19]. Computational models have confirmed that optimal mesh stiffness is location-specific and defect size-dependent and that pore geometry significantly influences stress distribution under tensile loading, findings with direct implications for the design of next-generation composite meshes [1].

Clinical outcomes

Laparoscopic Ventral and Incisional Hernia Repair

Laparoscopic ventral hernia repair is the principal clinical indication driving composite mesh development, given the mandatory intraperitoneal mesh positioning required by this approach. The laparoscopic technique reduces soft tissue dissection, wound complications, and hospital stay compared with open repair, and prosthetic mesh reinforcement significantly reduces recurrence compared with suture repair alone [4], which carries incisional hernia recurrence rates approaching 20% following midline laparotomy [12].

Composite mesh prostheses for laparoscopic ventral hernia repair have been evaluated in prospective multi-center trials demonstrating low recurrence rates and acceptable complication profiles. A systematic review and meta-analysis comparing biological versus synthetic mesh in ventral hernia repair found no significant differences in long-term recurrence rates between mesh types, though synthetic composite mesh was associated with lower overall costs and comparable infection rates even in contaminated operative fields [20]. Randomized trial evidence comparing lightweight composite mesh to conventional polypropylene or polyester mesh for incisional hernia repair showed similar primary outcomes for both approaches, with a non-significant trend toward increased recurrence with certain lightweight designs in larger defects [3].

Inguinal Hernia Repair

In inguinal hernia repair, composite and partially absorbable meshes have been investigated as alternatives to standard heavyweight polypropylene. Lightweight, large-pore meshes, including hybrid partially absorbable designs such as Ultrapro (polypropylene/polyglecaprone), demonstrate improved patient-reported outcomes for chronic groin pain and foreign body sensation without increasing recurrence, as reviewed by Earle and Mark [21]. In a multi-center randomized controlled trial, Xue et al. compared a non-crosslinked composite extracellular matrix graft derived from porcine urinary bladder matrix and small intestinal submucosa with lightweight synthetic mesh in laparoscopic inguinal hernia repair. At the four-year follow-up, the study reported equivalent hernia repair effectiveness, with no infection, seroma, or recurrence in the biological composite arm [22].

Complications and Safety Profile

The United States Food and Drug Administration has reported hernia recurrence rates up to 11%, surgical site infections in up to 21% of cases, and chronic pain in 0.3-68% of patients across hernia mesh types [1]. These broad ranges reflect the heterogeneity of patient populations, operative approaches, defect characteristics, and mesh designs included in available studies.

Adhesion formation remains the primary justification for composite mesh design. Standard polypropylene placed intraperitoneally without a barrier carries a small bowel resection rate of 20% at reoperation [7]. Composite barrier designs have demonstrably reduced adhesion burden. The superiority of permanent over absorbable barriers for long-term adhesion prevention has not been conclusively established, as clinically significant adhesions typically form during the early postoperative period before most barriers have degraded [12].

Mesh infection is a serious complication that frequently necessitates explantation. Polypropylene-based composite meshes can sometimes be salvaged with irrigation and drainage; ePTFE meshes virtually always require removal due to their microporous architecture that permits bacterial colonization while impeding macrophage-mediated clearance [8]. Multifilament structural meshes carry greater infection risk than monofilament equivalents due to bacterial harboring within interfilament spaces [2].

**Figure 5:**
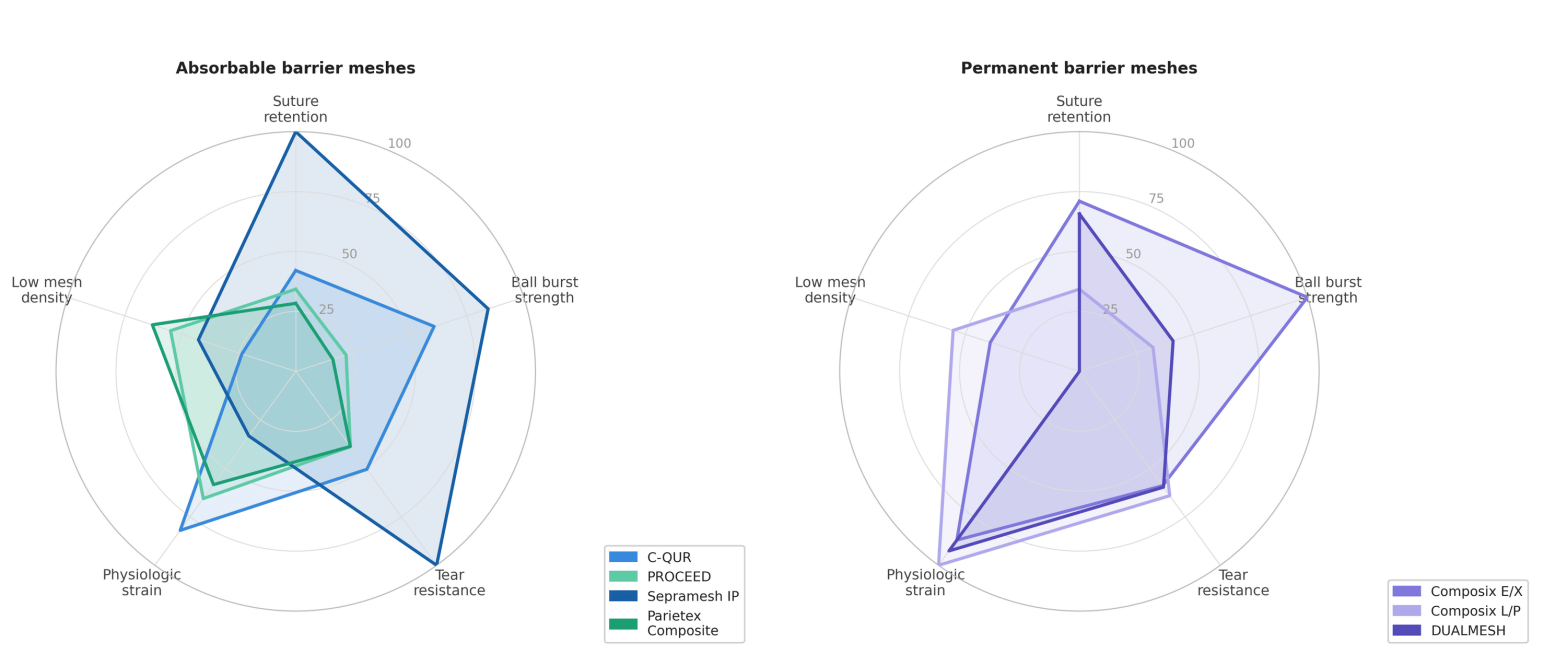
Multi-property normalized radar comparison of composite meshes Radar charts comparing normalized physicochemical performance scores (0-100) for absorbable barrier meshes (left panel) and permanent barrier meshes (right panel). Each metric is normalized to the maximum observed value. The "Low mesh density" axis is inverted (higher score = lighter mesh). Figure adapted from Deeken et al. [12] and created using GraphPad Prism (GraphPad Software, Boston, Massachusetts, United States)

Chronic pain is the most consequential long-term complication after inguinal hernia repair, with incidence reported between 10% and 30% in large registry studies [13]. Mesh-related fibrosis, shrinkage of up to 30% dimensional reduction with some designs, and perineural entrapment contribute to this phenomenon. Reducing mesh weight and increasing pore size are consistently associated with lower rates of chronic pain and better abdominal wall compliance in both experimental and clinical studies [15,16]. Seroma formation occurs in 5-15% of laparoscopic ventral hernia repair cases, predisposed by dead-space creation and lymphatic disruption; microporous meshes worsen seroma rates by limiting fluid resorption [12]. Mesh erosion into adjacent bowel, bladder, or vas deferens, while rare, represents a catastrophic late complication requiring prompt surgical management [13]. A normalized radar comparison of the key physicochemical properties of absorbable and permanent barrier meshes is provided in Figure 5.

Future directions and emerging innovations

Research in composite mesh technology continues along several parallel trajectories. Drug-eluting meshes represent a particularly promising frontier [19]. By embedding antibiotics, anti-inflammatory agents, or growth factors directly into mesh fibers or coatings, localized pharmacotherapy can be delivered at the implant site during the critical early healing period. Antibiotic-eluting meshes may substantially reduce surgical site infections in contaminated fields, while anti-inflammatory coatings may attenuate the foreign body reaction and reduce chronic pain without systemic side effects [1].

Auxetic mesh geometries, structures exhibiting a negative Poisson's ratio that expand laterally when stretched longitudinally, offer theoretical advantages in distributing mechanical stress more evenly across the mesh surface and improving fatigue resistance under cyclical loading [1]. Three-dimensional printing and patient-specific implant fabrication offer the prospect of meshes precisely tailored to an individual patient's defect geometry and tissue mechanical properties, enabling the optimization of both structural performance and tissue integration through computationally informed pore architectures [1].

Biodegradable and biosynthetic meshes are gaining traction as alternatives for contaminated fields or younger patients in whom permanent implant material is undesirable [19]. Biosynthetic constructs degrade over 12-36 months while supporting neotissue formation, bridging the gap between traditional biological scaffolds and permanent synthetic meshes. Standardization of physicochemical testing protocols and development of a common classification language between surgeons and biomedical engineers remains an outstanding unmet need, as emphasized by Deeken et al. [12].

## Conclusions

Composite meshes represent a significant and clinically meaningful advance over single-layer prosthetics in hernia repair, particularly when intraperitoneal mesh placement is required. By combining a structural reinforcement layer with an anti-adhesion barrier, these prostheses address the longstanding challenge of achieving durable abdominal wall support while minimizing visceral complications. However, substantial heterogeneity exists among commercially available composite meshes with respect to pore size, filament diameter, mesh density, suture retention strength, tear resistance, anisotropy, and physiologic strain behavior; as a result, no currently available composite mesh fulfils all the characteristics of an ideal prosthetic, and optimal outcomes depend not only on mesh design but also on careful patient selection, appropriate clinical application, and correct mesh orientation relative to the anisotropic demands of the native abdominal wall.

Emerging innovations, including drug-eluting surfaces, auxetic mesh geometries, three-dimensional printing, and biodegradable hybrid constructs, hold considerable promise in narrowing the gap between currently available prostheses and the ideal mesh for hernia repair. Continued collaboration between surgeons, biomaterials scientists, and biomedical engineers will be essential to translate these advances into safer, more durable, and more physiologically compatible implant designs.
